# Supplementary material for: The aetiological relationship between depressive symptoms and health-related quality of life: A population-based twin study in Sri Lanka
Source: PLoS One. 2022 Mar 30;17(3):e0265421. doi: 10.1371/journal.pone.0265421 (PMC8967029; doi:10.1371/journal.pone.0265421)
Supplement: S1 Table — (DOCX) [file pone.0265421.s001.docx]

**S1 Table.** Sex differences in means **(**Welch’s T-tests) for the SF-36 scales

| **Variable** | **Mean Score**  **Males** | **Mean Score**  **Females** | **t-value** | **df** | **p-value** | **95%CI** |
| --- | --- | --- | --- | --- | --- | --- |
| **General**  **Health** | 62.42 | 59.59 | 5.49 | 3706.3 | **<.001** | 1.82, 3.84 |
| **Emotional**  **Wellbeing** | 79.97 | 76.85 | 6.16 | 3737.9 | **<.001** | 2.13, 4.11 |
| **Energy/Fatigue** | 73.95 | 72.80 | 2.11 | 3601.8 | **.003** | .08, 2.22 |
| **Pain** | 88.72 | 85.10 | 5.61 | 3691.6 | **<.001** | 2.36, 4.90 |
| **Physical**  **Functioning** | 92.34 | 86.90 | 9.18 | 3791 | **<.001** | 4.28, 6.60 |
| **Social**  **Functioning** | 90.29 | 88.66 | 2.60 | 3612.9 | **.009** | .40, 2.86 |
| **Role**  **Physical** | 84.98 | 79.62 | 4.69 | 3703.3 | **<.001** | 3.11, 7.60 |
| **Role**  **Emotional** | 89.36 | 85.01 | 4.40 | 3753.7 | **<.001** | 2.41, 6.29 |
