# Supplementary material for: The aetiological relationship between depressive symptoms and health-related quality of life: A population-based twin study in Sri Lanka
Source: PLoS One. 2022 Mar 30;17(3):e0265421. doi: 10.1371/journal.pone.0265421 (PMC8967029; doi:10.1371/journal.pone.0265421)
Supplement: S2 Table — (DOCX) [file pone.0265421.s002.docx]

**S2 Table.** Within-twin cross-trait correlations of Depressive Symptoms and SF-36 scales

| **Variable** | **Males** | **Females** |
| --- | --- | --- |
| **General Health** | **-.30**  **(-.34/-.25)** | **-.29**  **(-.33/-.25)** |
| **Emotional Wellbeing** | **-.55**  **(-.58/-.51)** | **-.57**  **(-.60/-.54)** |
| **Energy/Fatigue** | **-.41**  **(-.45/-.37)** | **-.46**  **(-.49/-.42)** |
| **Pain** | **-.35**  **(-.40/-.31)** | **-.34**  **(-.38/-.31)** |
| **Physical Functioning** | **-.29**  **(-.34/-.25)** | **-.27**  **(-.31/-.23)** |
| **Social Functioning** | **-.38**  **(-.42/-.33)** | **-.38**  **(-.41/-.34)** |
| **Role Physical** | **-.30**  **(-.35/-.26)** | **-.31**  **(-.35/-.27)** |
| **Role Emotional** | **-.45**  **(-.49/-.41)** | **-.44**  **(-.48/-.41)** |
