# Supplementary material for: The aetiological relationship between depressive symptoms and health-related quality of life: A population-based twin study in Sri Lanka
Source: PLoS One. 2022 Mar 30;17(3):e0265421. doi: 10.1371/journal.pone.0265421 (PMC8967029; doi:10.1371/journal.pone.0265421)
Supplement: S3 Table — MZM: monozygotic males; MZF: monozygotic females; DZM: dizygotic males; DZF: dizygotic females; DZOS: dizygotic opposite sex twins. Significant correlations are shown in bold. (DOCX) [file pone.0265421.s003.docx]

**S3 Table.** Cross-twin cross-trait correlations of Depressive Symptoms with each SF-36 scale in same and opposite sex twin pairs (with 95% CIs)

| **Variable** | **MZM** | **DZM** | **MZF** | **DZF** | **DZOS** |
| --- | --- | --- | --- | --- | --- |
| **General**  **Health** | -.05  (-.14/.06) | .00  (-.11/.13) | **-.18**  **(-.25/-.10)** | -.04  (-.13/.05) | .04  (-.03/.12) |
| **Emotional**  **Wellbeing** | **-.19**  **(-.28/-.08)** | -.08  (-.22/.07) | **-.21**  **(-.29/-.12)** | **-.15**  **(-.25/-.04)** | -.01  (-.10/.07) |
| **Energy/**  **Fatigue** | **-.16**  **(-.26/-.05)** | -.03  (-.16/.11) | **-.20**  **(-.27/-.11)** | **-.15**  **(-.24/-.04)** | -.03  (-.11/.05) |
| **Pain** | **-.13**  **(-.23/-.03)** | -.11  (-.22/.02) | **-.17**  **(-.24/-.09)** | **-.12**  **(-.21/-.03)** | **-.03**  **(-.12/-.05)** |
| **Physical**  **Functioning** | -.12  (-.24/.02) | **-.20**  **(-.32/-.06)** | **-.17**  **(-.24/-.10)** | **-.15**  **(-.25/-.05)** | -.06  (-.14/.02) |
| **Social**  **Functioning** | **-.21**  **(-.32/-.08)** | -.17  (-.31/.00) | **-.20**  **(-.30/-.10)** | **-.15**  **(-.26/-.02)** | -.09  (-.19/.01) |
| **Role Physical** | -.08  (-.18/.04) | .00  (-.12/.14) | **-.18**  **(-.26/-.10)** | **-.20**  **(-.29/-.10)** | -.04  (-.12/.04) |
| **Role Emotional** | -.11  (-.22/.01) | -.05  (-.19/.10) | **-.20**  **(-.28/-.12)** | **-.21**  **(-.30/-.11)** | -.07  (-.15/.01) |

Note: *MZM*: monozygotic males; *MZF*: monozygotic females; *DZM*: dizygotic males; *DZF*: dizygotic females; *DZOS*: dizygotic opposite sex twins. Significant correlations are shown in bold.
