# Supplementary material for: The aetiological relationship between depressive symptoms and health-related quality of life: A population-based twin study in Sri Lanka
Source: PLoS One. 2022 Mar 30;17(3):e0265421. doi: 10.1371/journal.pone.0265421 (PMC8967029; doi:10.1371/journal.pone.0265421)
Supplement: S4 Table — The best fitting models are indicated in bold. Sat: saturated phenotypic correlation model; Sub1: constrained correlation model; HetACE: quantitative heterogeneity model testing for quantitative sex differences in additive genetic variance (A), shared environment variance (C), and non-shared environment variance (E); ScACE: scalar model, model where variances are allowed to differ across sexes by a constant multiplier; HomACE: homogeneity model, where the standardised ACE parameters are equated across sex; -2LL: minus twice the log of the likelihood of the data; df: degrees of freedom; ΔLL(Δdf): the difference in -2LL and df of two models which is χ2 distributed. AIC: Akaike’s Information Criterion. (DOCX) [file pone.0265421.s004.docx]

**S4 Table.** Univariate ACE model-fit statistics of Depressive Symptoms and SF-36 scales.

| Variable | **MODEL** | **ep** | **-2LL** | **df** | **AIC** | **ΔLL(Δdf)** | **p-value** |
| --- | --- | --- | --- | --- | --- | --- | --- |
| Depressive  Symptoms | Sat | 9 | 10355.61 | 3830 | 2695.61 | - | - |
|  | Sub1 | 8 | 10444.66 | 3831 | 2782.66 | 89.05(1) | 3.85E-21 |
|  | HetACE | 8 | 10355.73 | 3831 | 2693.73 | - | - |
|  | **ScACE** | **6** | **10358.99** | **3833** | **2692.99** | **3.26 (2)** | **.20** |
|  | HomACE | 5 | 10451.93 | 3834 | 2783.93 | 96.20(3) | 1.02E-20 |
| General  Health | Sat | 9 | 14416.67 | 3847 | 6722.67 | - | - |
|  | Sub1 | 8 | 14441.33 | 3848 | 6745.33 | 24.66(1) | 6.83E-7 |
|  | **HetACE** | **8** | **14418.50** | **3848** | **6772.50** | **-** | **-** |
|  | ScACE | 6 | 14425.37 | 3850 | 6725.37 | 6.87(2) | .003 |
|  | HomACE | 5 | 14448.76 | 3851 | 6746.76 | 3.25(3) | 1.22E-6 |
| Energy/  Fatigue | Sat | 9 | 11752.72 | 3847 | 4058.72 | - | - |
|  | Sub1 | 8 | 11754.83 | 3848 | 4058.83 | 2.11(1) | .15 |
|  | HetACE | 8 | 11752.96 | 3848 | 4056.96 | - | - |
|  | ScACE | 6 | 11754.86 | 3850 | 4054.86 | 1.91(2) | .39 |
|  | **HomACE** | **5** | **11756.50** | **3851** | **4054.50** | **3.54(3)** | **.32** |
| Emotional Wellbeing | Sat | 9 | 14415.11 | 3847 | 6721.11 | - | - |
|  | Sub1 | 8 | 14432.29 | 3848 | 6736.29 | 17.18(1) | 3.41E-5 |
|  | HetACE | 8 | 14415.39 | 3848 | 6719.40 | - | - |
|  | **ScACE** | **6** | **14417.66** | **3850** | **6717.66** | **2.27(2)** | **.32** |
| Pain | HomACE | 5 | 14434.05 | 3851 | 6732.05 | 18.65(3) | <.001 |
|  | Sat | 9 | 17986.93 | 3848 | 10290.93 | - | - |
|  | Sub1 | 8 | 18002.20 | 3849 | 10304.20 | 15.26(1) | 9.35E-5 |
|  | HetACE | 8 | 17994.79 | 3849 | 10296.79 | - | - |
|  | **ScACE** | **6** | **17997.04** | **3851** | **10295.04** | **2.25(2)** | **.33** |
|  | HomACE | 5 | 18012.94 | 3852 | 10308.94 | 18.15(3) | <.001 |
| Physical functioning | Sat | 9 | 11809.19 | 3847 | 4115.19 | - | - |
|  | Sub1 | 8 | 11840.96 | 3848 | 4144.96 | 31.77(1) | 1.73E-08 |
|  | HetACE | 8 | 11814.39 | 3848 | 4118.39 | - | - |
|  | **ScACE** | **6** | **11818.52** | **3850** | **4118.52** | **4.13(2)** | **.13** |
|  | HomACE | 5 | 11851.09 | 3851 | 4149.09 | 36.69(3) | 5.34E-08 |
| Role  Emotional | Sat | 9 | 14263.04 | 3847 | 6569.04 | - | - |
|  | Sub1 | 8 | 14284.18 | 3848 | 6588.18 | 21.14(1) | 4.27E-06 |
|  | **HetACE** | **8** | **14265.77** | **3848** | **6569.77** | **-** | **-** |
|  | ScACE | 6 | 14272.17 | 3850 | 6572.17 | 6.40(2) | .041 |
|  | HomACE | 5 | 14292.23 | 3851 | 6590.23 | 26.46(3) | 7.63E-06 |
| Role Physical | Sat | 9 | 20608.09 | 3847 | 12914.09 | - | - |
|  | Sub1 | 8 | 20626.69 | 3848 | 12930.69 | 18.60(1) | 1.61E-05 |
|  | **HetACE** | **8** | **20610.31** | **3848** | **12914.31** | **-** | **-** |
|  | ScACE | 6 | 20626.17 | 3850 | 12926.17 | 15.86(2) | <.001 |
|  | HomACE | 5 | 20639.55 | 3851 | 12937.55 | 29.24(3) | 1.99E-06 |
| Social Functioning | Sat | 9 | 4185.07 | 3888 | -3590.93 | - | - |
|  | Sub1 | 8 | 4185.61 | 3889 | -3592.39 | .54(1) | .46 |
|  | **HetACE** | **9** | **4187.21** | **3890** | **-3592.79** | **-** | **-** |
|  | HomACE | 6 | 4375.23 | 3893 | -3410.77 | 188.02(3) | 1.63E-40 |

Note: *Sat:* saturated phenotypic correlation model; *Sub1*: constrained correlation model; *HetACE*: quantitative heterogeneity model testing for quantitative sex differences in additive genetic variance *(A)*, shared environment variance *(C)*, and non-shared environment variance (E); *ScACE*: scalar model, model where variances are allowed to differ across sexes by a constant multiplier; *HomACE*: homogeneity model, where the standardised ACE parameters are equated across sex; -*2LL*: minus twice the log of the likelihood of the data; *df:* degrees of freedom; *ΔLL(Δdf)*: the difference in -2LL and df of two models which is *χ*^2^ distributed. *AIC*: Akaike’s Information Criterion; The best fitting models are indicated in bold.
