# Supplementary material for: The aetiological relationship between depressive symptoms and health-related quality of life: A population-based twin study in Sri Lanka
Source: PLoS One. 2022 Mar 30;17(3):e0265421. doi: 10.1371/journal.pone.0265421 (PMC8967029; doi:10.1371/journal.pone.0265421)
Supplement: S5 Table — The best fitting models are indicated in bold. Sat = saturated phenotypic model; Scalar = model where variances are allowed to differ across sexes by a constant multiplier; Scalar HetACE = hybrid scalar-heterogeneity ACE model testing for quantitaive sex differences only by accounting for sex variance differences in depressive symptoms (scalar variable); Scalar QualA, Scalar QualC = hybrid scalar-heterogeneity ACE models testing for quallitative sex differences in additive genetic variance (A) and shared environment variance (C) respectively, by accounting for sex variance differences in depressive symptoms; HomACE = homogeneity model where the standardised ACE parameters are equated across sex; Scalar HomACE = hybrid scalar-homogeneity ACE model where the standardised ACE parameters are equated across sex, allowing for sex variance differences in depressive symptoms. The Scalar QualA, Scalar QualC, and HomACE models were compared to the Scalar HetACE model. For variables with a Scalar HomACE or a Scalar model only, we did not fit heterogeneity models because there were no aetiological differences across sexes identified in the univariate analyses. -2LL: minus twice the log of the likelihood of the data; df: degrees of freedom; ΔLL(Δdf): the difference in -2LL and df of two models which is χ2 distributed. AIC: Akaike’s Information Criterion. (DOCX) [file pone.0265421.s005.docx]

**S5 Table.** Bivariate ACE model-fit statistics of Depressive Symptoms with each SF-36 scale

| Variable | **MODEL** | **ep** | **-2LL** | **df** | **AIC** | **ΔLL(Δdf)** | **p-value** |
| --- | --- | --- | --- | --- | --- | --- | --- |
| General  Health | Sat | 25 | 24409.45 | 7670 | 9069.46 | - | - |
|  | **Scalar**  **HetACE** | **20** | **24420.18** | **7675** | **9070.18** | - | - |
|  | Scalar  Qual A | 24 | 24413.13 | 7671 | 9071.13 | 7.31(4) | .12 |
|  | Scalar  Qual C | 24 | 24412.87 | 7671 | 9070.87 | 7.05(4) | .13 |
| Social Functioning | Sat | 25 | 14523.83 | 7711 | 14573.83 | - | - |
|  | **Scalar**  **HetACE** | **22** | **14527.89** | **7716** | **14571.89** | **-** | **-** |
|  | HomACE | 16 | 14529.00 | 7722 | 14561.00 | 1.11(6) | .98 |
| Role Physical | Sat | 25 | 30584.89 | 7670 | 15244.89 | - | - |
|  | **Scalar**  **HetACE** | **20** | **30593.46** | **7675** | **15243.46** | - | - |
|  | Scalar  Qual A | 24 | 30589.83 | 7671 | 15247.83 | 3.62 (4) | .46 |
|  | Scalar  Qual C | 24 | 30587.94 | 7671 | 15245.94 | 5.52 (4) | .24 |
| Role Emotional | Sat | 25 | 23776.33 | 7670 | 8436.33 | - | - |
|  | **Scalar**  **HetACE** | **20** | **23785.42** | **7675** | **8435.42** | - | - |
|  | Scalar  Qual A | 24 | 23780.40 | 7671 | 8438.40 | 5.93(4) | .20 |
|  | Scalar  Qual C | 24 | 23779.48 | 7671 | 8437.48 | 5.02(4) | .29 |
| Emotional  Wellbeing | Sat | 25 | 23309.11 | 7670 | 7969.11 | - | - |
|  | **Scalar** | **15** | **23317.32** | **7680** | **7957.32** | **8.21(10)** | **.61** |
| Energy/  Fatigue | Sat | 25 | 21295.44 | 7670 | 5955.44 | - | - |
|  | **Scalar**  **HomACE** | **14** | **21307.90** | **7681** | **5945.90** | **12.45(11)** | **.33** |
| Pain | Sat | 25 | 27853.87 | 7671 | 12511.87 | - | - |
|  | **Scalar** | **15** | **27869.28** | **7681** | **12507.28** | **15.41(10)** | **.12** |
| Physical  Functioning | Sat | 25 | 21867.24 | 7670 | 6527.24 | - | - |
|  | **Scalar** | **15** | **21882.51** | **7680** | **6522.51** | **15.27(10)** | **.12** |

Note: *Sat=* saturated phenotypic model*; Scalar=* model where variances are allowed to differ across sexes by a constant multiplier; *Scalar HetACE=* hybrid scalar-heterogeneity ACE model testing for quantitaive sex differences only by accounting for sex variance differences in depressive symptoms (scalar variable); *Scalar QualA, Scalar QualC=* hybrid scalar-heterogeneity ACE models testing for quallitative sex differences in additive genetic variance *(A)* and shared environment variance *(C)* respectively, by accounting for sex variance differences in depressive symptoms; *HomACE*= homogeneity model where the standardised ACE parameters are equated across sex; *Scalar HomACE=* hybrid scalar-homogeneity ACE model where the standardised ACE parameters are equated across sex, allowing for sex variance differences in depressive symptoms. The Scalar QualA, Scalar QualC, and HomACE models were compared to the Scalar HetACE model. For variables with a Scalar HomACE or a Scalar model only, we did not fit heterogeneity models because there were no aetiological differences across sexes identified in the univariate analyses. -*2LL*: minus twice the log of the likelihood of the data; *df:* degrees of freedom; *ΔLL(Δdf)*: the difference in -2LL and df of two models which is *χ*^2^ distributed. *AIC*: Akaike’s Information Criterion; The best fitting models are indicated in bold.
