# Supplementary material for: The aetiological relationship between depressive symptoms and health-related quality of life: A population-based twin study in Sri Lanka
Source: PLoS One. 2022 Mar 30;17(3):e0265421. doi: 10.1371/journal.pone.0265421 (PMC8967029; doi:10.1371/journal.pone.0265421)
Supplement: S6 Table — Note: (1/1) and (-1/-1): model did not converge to estimate the 95%CI; Variables fit to a scalar-scalar model (*) or a scalar-homogeneity model (**) do not have a male/female breakdown because the (standardized) ACE parameters are equated across sexes, and either the variances are allowed to differ by a constant multiplier (scalar), or not (homogeneity model); M = Male, F = Female. (DOCX) [file pone.0265421.s006.docx]

**S6 Table.** Genetic (*Ra*), shared-environmental (*Rc*), and non-shared environmental (*Re*) correlations between Depressive Symptoms and SF-36 scales

| **Variable** | **Sex** | **Ra (95%CI)** | **Rc (95%CI)** | **Re (95%CI)** |
| --- | --- | --- | --- | --- |
| **General Health** | M | -.46 (-1/.02) | **1 (1//1)** | **-.37 (-.47/-.26)** |
|  | F | **-1 (-1/-1)** | .14 (-1/1) | **-.21 (-.29/-.12)** |
| **Social Functioning** | M | -.79 (-1/1) | -.55 (-1/1) | **-.37 (-.52/-.22)** |
|  | F | -1 (-1/1) | -1 (-1/-1) | **-.45 (-.55/-.31)** |
| **Role Physical** | M | -.95 (-1/.05) | 1 (1/1) | **-.30 (-.41/-.20)** |
|  | F | -.58 (-1/.25) | **-1 (-1/-1)** | **-.17 (-.27/-.07)** |
| **Role Emotional** | M | **-.47 (-.84/-.13)** | 1 (1/1) | **-.46 (-.54/-.36)** |
|  | F | -1 (-1/1) | -1 (-1/-1) | **-.32 (-.40/-.24)** |
| ***Emotional Wellbeing** | M\|F | **-.90 (-1/-.52)** | 1 (1/1) | **-.53 (-.58/-.47)** |
| ****Energy/Fatigue** | M\|F | **-1 (-1/-1)** | 1 (-1/1) | **-.38 (-.44/-.32)** |
| ***Pain** | M\|F | -1 (-1/-1) | -.94 (-1/1) | **-.27 (-.33/-.20)** |
| ***Physical Functioning** | M\|F | -.44 (-1/.69) | -1 (-1/-1) | **-.18 (-.26/-.10)** |

Note: (1/1) and (-1/-1): model did not converge to estimate the 95%CI; Variables fit to a scalar-scalar model **(*)** or a scalar-homogeneity model **(**)** do not have a male/female breakdown because the (standardized) ACE parameters are equated across sexes, and either the variances are allowed to differ by a constant multiplier (scalar), or not (homogeneity model); M=Male, F= Female
