# Supplementary material for: The aetiological relationship between depressive symptoms and health-related quality of life: A population-based twin study in Sri Lanka
Source: PLoS One. 2022 Mar 30;17(3):e0265421. doi: 10.1371/journal.pone.0265421 (PMC8967029; doi:10.1371/journal.pone.0265421)
Supplement: S7 Table — (DOCX) [file pone.0265421.s007.docx]

**Data Dictionary CoTaSS**

| **Variable** | **Variable explanation** | **Values** |
| --- | --- | --- |
| numtw.id | Family id |  |
| numtw.no | Twin number | 1= Singleton 2= Twin 3= Triplet |
| sing.or.twin | Singleton/Twin | 1 = Singleton 2 = Twin |
| age.yrs | Age in years |  |
| sex.c1c2 | Sex | 0 = Male 1= Female |
| c1c2.sexzyg | Group per sex and zygosity | 1=MZ males  2=DZ males  3=MZ females  4=DZ females  5=Opposite sex twins  6=Singleton males  7=Singleton females |
| pbecktot | Beck Depression Total score | 1-10 = These ups and downs are considered normal 11-16 = Mild mood disturbance 17-20 = Borderline clinical depression 21-30 = Moderate depression 31-40 = Severe depression > 40 = Extreme depression |
| sf.phys.func | SF-36 Physical functioning scale | 0 = More/max impairment 100 = Less/min impairment/no disability |
| sf.role.phys | SF-36 Role limitations due to physical health scale | 0 = Severely impaired by physical health problems 100 = Not impaired at all |
| sf.role.emo | SF-36 Role limitations due to emotional problems scale | 0 = Severely impaired by emotional health problems 100 = Not impaired at all |
| sf.enrg.fat | SF-36 Energy/fatigue/ vitality scale | 0 = Low energy, fatigue, low vitality 100 = Full of energy, no fatigue |
| sf.emo.wllb | SF-36 Emotional well-being scale | 0 = Low emotional wellbeing 100 = High/Good emotional wellbeing |
| sf.soc.func | SF-36 Social functioning scale | 0 = No social functioning 100 = Highly social |
| sf.pain | SF-36 Pain scale | 0 = High levels of pain 100 = No pain |
| sf.gen.hlth | SF-36 General health scale | 0 = Poor general health 100 = Good general health |
